# Close-canopy lighting, an effective energy-saving strategy for overhead sole-source LED lighting in indoor farming

**DOI:** 10.3389/fpls.2023.1215919

**Published:** 2023-07-27

**Authors:** Fatemeh Sheibani, Mike Bourget, Robert C. Morrow, Cary A. Mitchell

**Affiliations:** ^1^ Department of Horticulture and Landscape Architecture, Purdue University, West Lafayette, IN, United States; ^2^ Space Applications-Environmental Systems, Sierra Space, Madison, WI, United States

**Keywords:** close-canopy lighting, crop-canopy photon capture efficiency, energy utilization efficiency, LEDs, vertical farming, indoor farming, photon distribution, leafy greens

## Abstract

Significant advancement has been achieved improving electrical efficiency and photon efficacy of light-emitting diodes (LEDs) as the sole source of crop lighting for indoor farming. However, a significant portion of highly efficient photon emissions from improved LEDs is wasted by natural beam spread beyond cropping areas. Additional attention is needed to enhance crop-canopy photon capture efficiency (CCPCE), the fraction of photons emitted from LEDs actually incident upon foliar canopies. We postulate that by taking advantage of unique physical properties of LEDs, such as low radiant heat at photon-emitting surfaces and dimmable photon emissions, reduced vertical separation distance between light-emitting surfaces and light-receiving surfaces will enhance CCPCE by capturing more obliquely emitted photons that otherwise are lost. This “close-canopy-lighting” (CCL) strategy was tested in two ways: For an energy-efficiency strategy, LEDs were dimmed to the same photosynthetic photon flux density (PPFD) of 160 µmol m^-2^ s^-1^ at 45-, 35-, 25-, and 15-cm separation distances between lamps and cropping surfaces. For a yield-enhancement strategy, dimming was not applied, so higher PPFDs occurred at each separation distance closer than 45 cm for the same input energy. In the first strategy, the same biomass of lettuce (*Lactuca sativa* L. cv. Rouxai) was produced at each separation distance, while significantly lower energy was expended for lighting at each closer separation. Significantly higher biomass was produced at reduced separation distances with the same energy expenditure by LEDs using the yield-enhancement strategy. For both strategies, energy-utilization efficiency (g/kWh) doubled at the closest separation distance of 15 cm compared to the standard 45-cm separation distance. Even higher energy-utilization efficiency was achieved at a 25-cm separation distance when growth compartments were enclosed with a reflective curtain in the yield-enhancement strategy. Our findings suggest that CCL is a highly effective energy-saving strategy for overhead LED lighting, suggesting the need for innovative next-generation re-design of height-adjustable LED mounts and controlled air movement between tiers of indoor farms utilizing CCL.

## 1 Introduction

As one of the newer sectors of Controlled-Environment Agriculture (CEA), Vertical Farming (VF) is an innovative form of Indoor Farming (IF) in which plants typically are grown either hydroponically or aeroponically in vertical stacks. Light-emitting diodes (LEDs) are considered the best sole source of crop lighting in a warehouse setting.

Vertical farming is proliferating as demand for local, fresh, year-round produce rises, especially in seasonal, urban areas. Based on recent Indoor Farming market analysis, VF has a projected 25.7% compound annual growth rate from 2020 to 2027, which compares favorably with other CEA sectors in the United States (Grand View Research, 2020). Other benefits of IA/VF systems include not contributing to scarcity of arable farm land or freshwater resources (Benke and Tomkins, 2017). The risk of crop loss due to extreme weather conditions is avoided, and fossil-fuel use for transporting produce to urban markets is minimized. On the other hand, there are economic concerns regarding the VF industry. This emerging industry is typically considered expensive entrepreneurship due to high Capital Expenses (CAPEX) and significant Operational Expenses (OPEX). Two of the most important CAPEX components are expensive land in urban areas and the high initial cost of installing LED lighting systems (Qiu et al., 2020).

Electric lighting is one of the most expensive OPEX costs of vertical farming. Even though LEDs are more energy efficient than other electric lighting sources, sole-source electric lighting is still a major input cost, and seeking ways to save energy for lighting is a significant issue for commercial growers. Electricity typically accounts for 25-30% of total OPEX (Kozai, 2013; Kong et al., 2019; Kozai and Niu, 2020). Although the VF industry is expanding worldwide thanks to enthusiastic financial investors, high OPEX is keeping profitability potential of this nascent industry fragile and often elusive. As of 2019, based on a CEA Census report, only 37% of VFs were reported to be profitable (Global CEA Census Report, 2019). Although 58% of VFs reported to experience financial improvement based on the Global CEA Census Report (2021), indoor farms began ceasing operations in 2022 and 2023.

A cost-efficient lighting system is one of the most critical determinants of indoor-farming profitability (Davis & Burns, 2016; Cocetta et al., 2017). An efficient lighting system is a combination of both efficient fixtures as well as effective canopy photon capture (Nelson and Bugbee, 2014).

Over the past decade, notable success has been achieved improving LED electrical efficiency and photon efficacy (Mitchell and Sheibani, 2020). It has been estimated that these parameters are approaching their maximum theoretical values (Kusuma et al., 2020). *Electrical efficiency* is defined as the ratio of optical power output to electrical power input (WW^-1^). As of 2020, based on Kusuma et al. (2020), blue LEDs had a maximum theoretical efficiency of 0.93, followed by red (0.81), far-red (0.77), phosphor-converted cool-white (0.76), phosphor-converted warm-white (0.69), and green (0.42). On the other hand, photon efficacy is a metric to characterize the suitability of LED lighting systems for horticultural purposes. Photosynthetic efficacy is the ratio of micromoles of photosynthetic photons (400-700 nm) generated to the electrical energy applied (µmole J^-1^). In 2020, far-red LEDs had the highest photon efficacy (4.7), followed by red (4.5), blue (3.5), cool-white, warm-white, and green with photon efficacies of 2.9, 2.6, and 1.9 (µmole J^-1^), respectively (Kusuma et al., 2020).

Other advantages that make LEDs the choice for sole-source lighting include a significantly longer operational lifetime: They do not typically burn out. Instead, performance weakens gradually, and time of decline to 70% of its original intensity typically is 50,000 hours for horticultural LEDs (Kusuma et al., 2020; Paucek et al., 2020), at which time they are candidates for change.

Compared with other electric-lighting sources that have been used for plant growth, LEDs are not bulky, enabling flexibility of fixture design and straightforward installment and replacement. LEDs are solid-state devices that integrate easily with digital-control systems.

**Figure 1:**
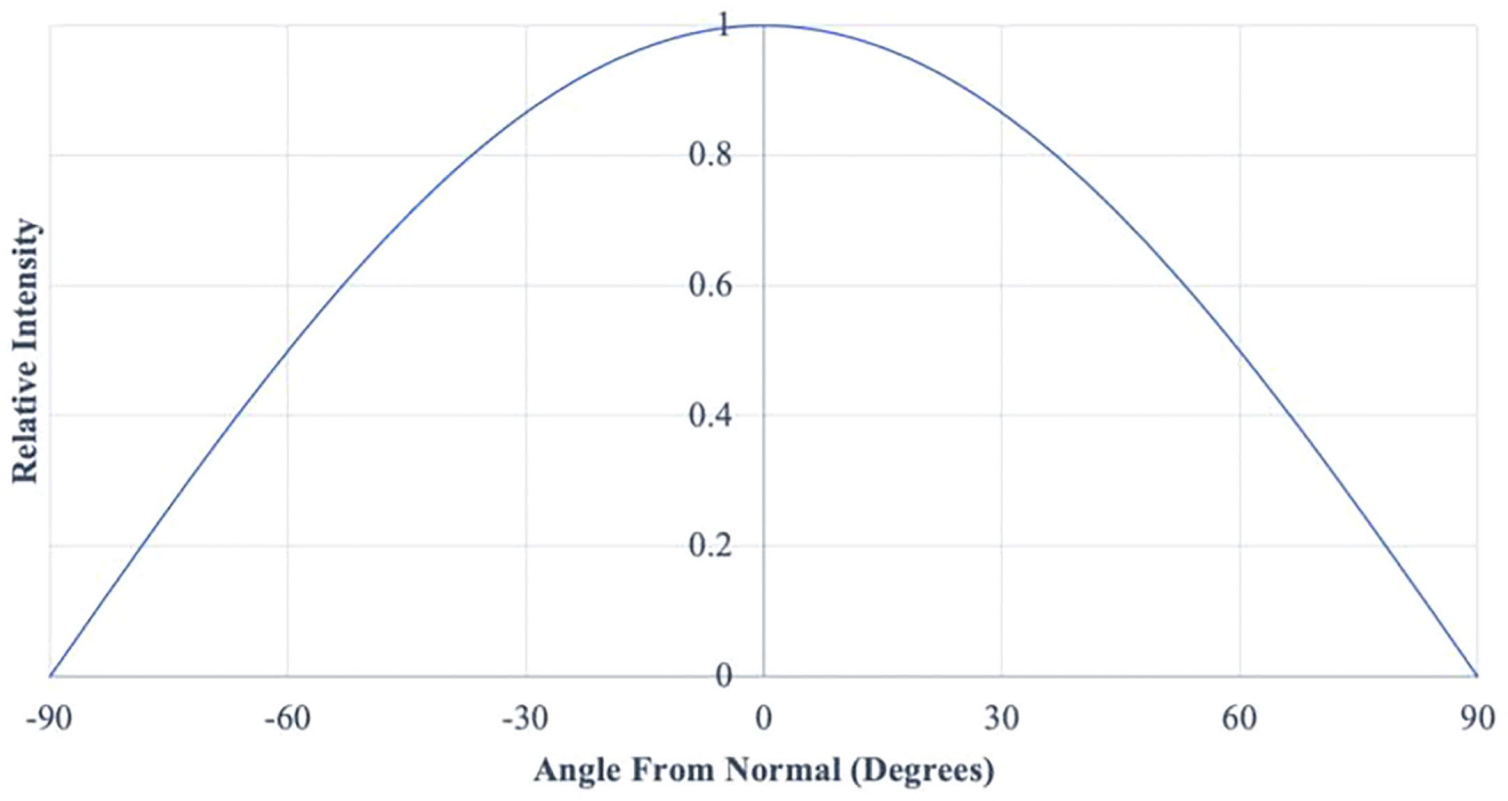
A Lambertian distribution of photon intensity from Lumi-LEDs used to construct the BPSE lighting system used in this study (figure description included in Result section) (LUMILEDS, 2023).

Studies of LED indoor illumination patterns have emphasized uniformity of intensity for human perception (Moreno & Tzonchev, 2004; Moreno et al., 2007; Yang et al., 2008; Sun & Lee, 2022). A generalized Lambertian function, estimated by Gaussian distribution, characterizes the typical geometric illumination pattern of LEDs (Yang et al., 2008; Sun and Lee, 2022). Although the illumination pattern of a Lambertian distribution is 180°, 87% of that pattern occurs in a half-width of ± 60°C and a view angle of 120 ° (**Figure 1**). For indoor crop lighting, an LED lighting source can be a “bulb-type” emitter or a planar/filamentous array positioned horizontally (overhead) or vertically (intra-canopy) (Tsao et al., 2015; Cocetta et al., 2017). As a recently-adopted technology for indoor crop lighting, available LED choices also follow that general Lambertian distribution (Stutte, 2009; Nicole et al., 2016; Lee et al., 2023). The relative intensity of photon emissions taken from a data sheet for the LED arrays used for the present study was plotted by the authors from a data sheet for the Lumi-LED LEDs used by the Orbital Technologies Corporation (ORBITEC, now Sierra Space), to construct the Biomass-Production-System-for-Education (BPSE) adjustable-height LED arrays and was found to be Lambertian in nature (**Figure 1**). If intensity at 0^0^ angle from normal is 160 μmol/m^2^/s (as per this study), then 80 μmol/m^2^/s would be measured by a cosine-corrected light sensor at ± 60^0^ (a cone) with further decreasing relative intensities extending to 90^0^ (a hemisphere) in all directions.

Although “beam spread” or “view angle” of individual LED packages is available in manufacturer catalogs and specification sheets, there is not enough working knowledge about how to best use the irradiation pattern of Lambertian LEDs as the sole-source for plant lighting, and the pattern of irradiation when multiple packages are installed in either bulb or filament type is neither easy to estimate nor is widely reported. Inspection of several online catalogues offering LEDs for horticultural applications advertised view angles of 120^0^ to 130^0^.

In a vertical-farm setting, various lighting-installment plans have been used by growers. Depending on growth-compartment layout, tube-type LED modules may run along or across the growing area. In each case, an adequate number of LEDs must be installed to ensure uniformity of light intensity up to the edges of benches, thereby avoiding low-light-intensity edge effects and crop-growth gradients across benches.

Different factors affect irradiation pattern for crop-growth applications, including density of LED packages/modules on fixture undersurfaces, and separation distance between the LED lighting system and crop surface. In theory, LED packages are basically hemispherical radiators of light energy (radiating at 180°) after mounting on a flat surface for overhead-lighting applications, although most photons radiate within the 120° Lambertian beam spread. In that context, some photons radiate directly downward toward cropping surfaces, but also outward, beyond cropping surfaces at all angles up to maximum beam spread, leading to considerable loss of photon emissions beyond bench dimensions, especially when integrated over entire photoperiods and cropping cycles. Primary and secondary optics installation further shape beam angle, with accompanying energy-absorbance loss for every photon reflected off of a solid surface. A primary optic can be a protective lens made with epoxy or silicone resin to enhance the lifetime of LEDs by protecting them from moisture and dust (Massa et al., 2006; Alim et al., 2021). Moreover, an LED package also can be enclosed and protected by a “pit primary optic”. The pit might be covered with an encapsulation, into which a phosphor material is incorporated to create white radiation from blue LEDs (*Personal Communication, Elio Jin-Ha Kim, PhD, Samsung Corporation*, 2022). The wall-coating material within the pit typically is titanium dioxide (TiO2) because of its high reflectivity index (Silva et al., 2022).

Beam intensity decreases sharply beyond 120°-130° out to 180° in current LED module design for “bulb-type” emitters after installing a primary optic (OSRAM, 2021; Samsung, 2021). Secondary-optic lenses, such as a cylinder around each LED module, die, or engine, typically lined with reflective materials, sharply attenuate beam spread and are used to enhance spectral uniformity and intensity, as LEDs are brighter in the center of an attenuated beam (Kuo et al., 2011). Depending on secondary-optic lens design, beam spread may be cut to half the original viewing angle (Zhenrong et al., 2009). The energy cost of attenuating beam spread with secondary optics is reduced electrical efficiency and photon efficacy due to absorbance losses whenever photons reflect off of solid surfaces.

Among other LED advantages, low radiant heat production at photon-emitting surfaces allows LED fixtures to be placed close to crop canopies (Bourget, 2008; Massa et al., 2008; Singh et al., 2015; Davis & Burns, 2016). Another advantage is moderate dimmability without significant power loss (Pattison et al., 2016).

Present indoor-crop-lighting practices do not take full advantage of vastly improved LED electrical efficiency and photon efficacy because a significant portion of fixture light beams fall outside cropping areas, especially from LEDs mounted above or beyond bench edges, thereby negating many energy-use improvements that have been made in recent years.

**Figure 2:**
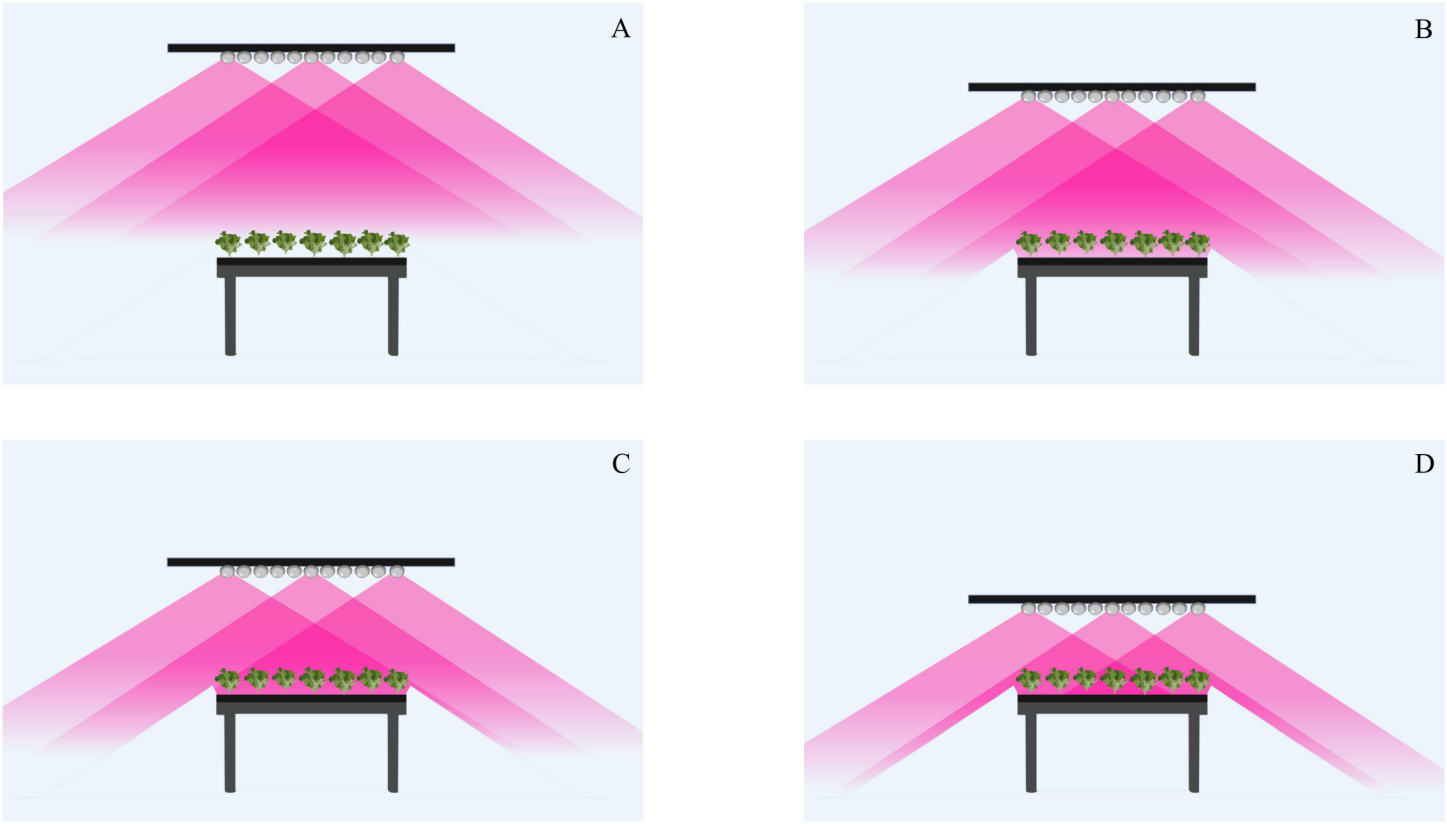
LED irradiation pattern at standard separation distance of 45 cm **(A)** compared with closer separation distances of 35 cm **(B)**, 25 cm **(C)**, 15 cm **(D)** with a standard beam spread of 120°. Only center and edge LED modules are depicted as energized for illustration. Created with BioRender.com.

By leveraging the low radiant heat and dimmability properties of LEDs, we postulated that reducing the vertical separation distance between lighting source and crop surface would enhance energy-use efficiency without heat-scorch damage to the crop. The “coolness” factor of LEDs positioned closer to plant tissues allows the electrical power needed to deliver a given light intensity to be tuned down compared to what would be required for a hot light source to achieve the same light intensity at a considerably larger separation distance. At closer separation distance, the chance of obliquely emitted photons escaping the growth compartment is reduced (**Figure 2**). At the standard separation distances used in vertical farms, photon loss is considerable as a result of wide-angled “beam spread” falling beyond growth-compartment boundaries. The current-driven LED property allows lower energy expenditure by dimmed LEDs still achieving the same photon intensities at closer separation. The minimum separation distance that can be used will be determined by LED spacing and resulting spectral-composition uniformity (Mitchell, 2015).

For the present project, we postulated that by taking advantage of unique physical properties of LEDs, crop-canopy photon-capture efficiency (CCPCE), equivalent to the light-engineering term “utilance”, can be enhanced, thereby improving much-needed energy-use efficiency of the indoor-farming sector. In this study, we characterized two close-canopy-lighting (CCL) scenarios: energy-efficiency and yield-enhancement. The laboratory conducting this CCL proof-of-concept study has a long history of stepwise, related investigations. The productivity/energy-saving advantages of allowing plants to grow up, around lights within a crop foliar canopy was initially demonstrated using low-wattage fluorescent kitchen lamps arrayed 3-dimensionally in growth compartments, providing “intra-canopy lighting” (ICL) to vertically growing plants (Frantz et al., 1998, Frantz et al, 2000; Frantz and Mitchell, 1997). Relative coolness of LEDs at light-emitting surfaces and moderate dimmability without major power loss made LEDs the undeniable choice for sole-source ICL (Massa et al., 2006; Massa et al., 2008), as well as supplemental ICL of high-wire tomatoes growing in greenhouses (Gomez et al., 2013; Gómez & Mitchell, 2016). Previous work from the PI’s lab also demonstrated the substantial energy savings of targeted vs. full-coverage overhead lighting of leaf lettuce (Poulet et al., 2014). The present study is a next-step systematic characterization of the productivity and/or energy-saving effects of close-canopy overhead lighting on densely seeded young lettuce plants with rapid canopy closure.

## 2 Materials and methods

The standard separation distance between the photon-emitting surface of LEDs and a standing crop in a vertical farm setting is 40-50 cm (Kubota, 2020). Considering 45 cm as a standard control separation distance, three closer separation distances of 35, 25, and 15 cm were tested and compared, using a typical 120° beam-spread angle of bulb-type Lumi-led LED modules.

### 2.1 Scenario 1: Energy-efficiency strategy

As separation distance decreased, LEDs were dimmed to achieve the same light intensity at the crop surface, thereby reducing instantaneous power draw and integrated energy consumption over time. We hypothesized that equivalent fresh and dry shoot biomass would be produced at the closer separation distances for lower energy expenditure.

### 2.2 Scenario 2: Yield-enhancement strategy

The second scenario was designed for the benefit of commercial operations using non-dimmable LED lighting systems. We postulated that, as separation distance decreases, significantly higher PPFD would occur at the crop surface, and higher fresh/dry biomass yield would occur for the same electrical energy input (kWh).

### 2.3 Combination of close-canopy lighting and reflective curtains

Since some photons still escape from the edges of growth compartments, even at the closest separation distances, a vertical reflective curtain surrounding the growth compartment would reflect some already-escaped photons back into the growth compartment. A reflective curtain should additionally enhance both crop yield as well as energy-use efficiency for crop lighting within an enclosed growth compartment in combination with either scenario of close-canopy lighting.

### 2.4 Controlled-environment conditions

Experiments were conducted in a walk-in growth chamber (EGC, Chagrin Falls, Ohio), where all environmental parameters were under precise control. The temperature was set to 23/22 ± 2°C during the day and night, respectively. Day and night relative humidities were 70/80%, respectively. CO_2_ was injected beginning 8 days after starting experiments, when the first set of true leaves started expanding. CO_2_ level was injected and maintained at 800 µmol mol^-1^ during the day and at 400 µmol mol^-1^ during the night.

### 2.5 Lighting system and lighting adjustment

Four BPSE (Biomass Production System for Education) LED lighting systems (ORBITEC/Sierra Space Corporation, Madison, WI) were placed on wire-mesh benches inside the growth chamber, and each unit was connected to a power/energy meter (Poniie, PN-2000). A 16-h photoperiod was set for all experiments from 0600 h to 2200 h. Light intensity and spectral composition were adjusted using a spectroradiometer (Black-Comet, StellarNet Inc., Tampa, FL). Five measurements were made under each LED panel, one in the center, followed by two at 22 cm along the length and two at 10 cm across the width from the center point, close to the short and long edges of each tray. The average of five values was similar to the center-point measurement. BPSE lighting units included three channels of dimmable blue, green, and red wavebands with peak wavelengths of 447.5 nm, 530 nm, and 627 nm, respectively. The fixture was not designed specifically for CCL, although intensity and spectral composition were uniform at the closest separation distance tested. The LED fixtures were mounted on a scissors jack that allowed continuous vertical height adjustment, which was required for setting different separation distances. Photosynthetic photon flux density (PPFD) was 160 µmol m^-2^ s^-1^ consisting of 82% red (132 ± 2 µmol m^-2^ s^-1^), 9% blue (14 ± 2 µmol m^-2^ s^-1^), and 9% green (14 ± 2 µmol m^-2^ s^-1^) light. Cumulative electrical energy (kWh) consumed for lighting was recorded daily, and total energy use for lighting was recorded upon termination of each cropping experiment.

### 2.6 Close-canopy-lighting set up

Each BPSE lighting unit included an LED panel mounted on a height-adjustable rack. The fixture dimensions were 27 (L) × 17 (W) × 4 (H) inches (or 68.5 L × 43.1 W × 10.1 H cm), accommodating one standard 10 × 20 inches (25.4 × 50.8 cm) growth tray on the wire-mesh bench supporting all BPSE units. In that configuration, tray positions were fixed, but lighting fixtures could be set closer to or farther away from tray surfaces. LED lighting systems were set to four different test separation distances including 45 cm (17.71 inches) as control and three test separations including 35 cm (13.77 inches), 25 cm (9.84 inches), and 15 cm (5.9 inches).

For the energy-efficiency scenario, the same PPFD of 160 µmol m^-2^ s^-1^ and the same spectral composition of 82% red, 9% blue, and 9% green light were used for each BPSE at each separation distance tested.

For the yield-enhancement scenario, PPFD and spectrum were the same as for the energy-efficiency scenario at all separation distances tested during the first 4 days after starting an experiment to avoid greenhouse-effect heating under humidification domes initially covering the growth trays. Starting from day 5, domes were removed and final test separation distances were set. PPFDs were measured as 160, 280, 350, and 480 µmol m^-2^ s^-1^ for the 45-cm, 35-cm, 25-cm, and 15-cm separation distances, respectively.

### 2.7 Combination of close-canopy lighting and reflective-curtain setup

In a separate set of experiments, all four BPSE LED lighting units were set at a CCL separation distance of 25 cm. Two randomly selected BPSE units were enclosed with vertical curtains of reflective polyethylene film (white facing inward, black facing outward), with vertical fringes cut to ensure air movement. The application of reflective curtains was investigated in combination with both CCL scenarios. The PPFD was set to 160 µmol m^-2^ s^-1^ for the energy-efficiency scenario and to 350 µmol m^-2^ s^-1^ for the yield-enhancement scenario with the same spectrum as mentioned previously.

All BPSE LED lighting units were equipped with energy meters, and cumulative energy consumption for lighting was recorded daily. Each experiment was replicated twice, with the reflective curtain setup rotated among lighting units between experiments to validate results while controlling for position effect.

### 2.8 Plant material and substrate

Red oakleaf lettuce (*Lactuca sativa* cv. Rouxai; Rijk Zwaan, De Lier, Netherlands) was used as the model crop for this project. A 50:50 (V/V) coco coir/perlite substrate was used to grow “lawns” of baby lettuce in a set of two stacked 10×20 inches (25.4 cm×50.8 cm) standard trays. An inner, lining tray with a mesh bottom was placed within an outer, un-meshed propagation tray (Bootstrap Farmers, Downingtown, PA, USA). Translucent plastic domes were placed over newly planted trays during the first 4 days of each experiment to keep humidity level high under domes to promote uniform germination and emergence. The commercially available fertigation solution used contained macro- and micro-fertilizer nutrients designed specifically for hydroponic lettuce cultivation (Fancy Lettuce, AmHydro, Arcata, CA, USA). Salts were dissolved in RO water to form a nutrient solution with an Electrical Conductivity (EC) of 1.2-1.4 µS cm^-1^ and a pH of 5.6-5.8 as measured by a portable EC and pH meter (HI 9813-6 pH/EC/TDS/C, Hanna Instrument GroChek, Woonsocket, RI). After dome removal on day 4, bottom fertigation was initiated and repeated on a daily basis with slightly different regimes for scenarios 1 and 2. For the energy-efficiency scenario, 400-ml nutrient solution was applied to the outer tray for each of the first 4 days after de-doming followed by 2 consecutive days applying 200 ml, a single day of 400 ml, followed by 200 ml for the duration of the 15-day cropping cycle. On the day of harvest (day 15), 200-ml nutrient solution was added before removing trays from the growth chamber. For the yield-enhancement scenario, in which higher light intensities occurred under closer separation distances, enhanced demand for water required that 400 ml of nutrient solution or water be applied on a daily basis, with the exception of first and last applications of 200 ml each. In both scenarios, trays were weighted on a daily basis after applying nutrient solution, and the difference between heaviest and lightest trays was compensated for using RO water applied to the lightest trays, which strategy helped to maintain trays at the same water status with the same amount of nutrients applied.

As an end-point growth parameter, harvested total shoot fresh biomass was weighed (PL602E, Mettler-Toledo, Columbus, OH) on the experimental basis of standard tray. Weighed shoot-tissue samples were placed in a paper bag and subjected to 5 days at 60 °C in a forced-air drying oven (Isoptemp 180L, Thermo-Fisher scientific, Waltham, MA) before dry biomass was measured and recorded.

### 2.9 “Lawn-of-baby-greens” production system

During early stages of lettuce-crop production, plants were small, foliar canopies open, and photons were not used efficiently. While re-spacing plants was not an option, considering the ultimate target size of plants (small baby greens), space between plants was minimized upon planting. In order to minimize photon waste and hasten foliar canopy closure to overhead lighting, we developed a “lawn-of-baby-lettuce” growing system. Targeting 15-day cropping cycles, at which time the baby stage is reached and when the first set of true leaves are developing and a second set are emerging, 84 seeds with a distance of 1.5 inches (3.81cm) between seeds and away from tray edges were planted in each tray. Approximately 3200 ml of soilless substrate was formed into a leveled layer to 1.5 inches (3.81cm) depth in each inner, mesh tray. Propagation-meshed-bottom trays were placed into an unmeshed propagation tray filled with 2000 ml of half-strength nutrient solution for 5 min, after which excess nutrient solution was poured off by holding the inner tray at a 45° angle until dripping halted. Mesh trays were then placed within unmeshed trays without nutrient solution, and pelleted seeds were planted on the wetted surface using a custom-made Plexiglas seeding template. Seeds were compressed gently into the moistened medium using a solid plate (without holes) to ensure adequate moisture absorption and uniform germination.

### 2.10 Experimental design

The experimental layout was a randomized block design, in which each standard tray was considered as an experimental unit. Block refers to the area with homogenous environmental parameters, which is the delineated area under fixture arrays in this study. Each energy-efficiency and yield-enhancement test strategy was replicated four times, with separation distances rotated under various BPSE fixtures to correct for any fixture or position effects and to ensure data accuracy.

### 2.11 Statistical analysis

Data were subjected to one-way analysis of variance (ANOVA) using RStudio software (RStudio 1.2.5042, ^©^ 2009-2020 RStudio, Inc.) within R statistical package (R Core Team, 2020). Tukey’s HSD test at p-value < 0.05 was used to determine differences between means when applicable.

## 3 Results

**Figure 3:**
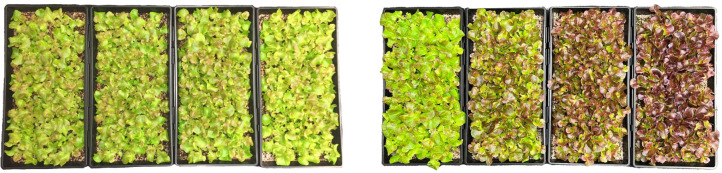
Lawn of baby-green production system in energy-efficiency scenario (left) and yield-enhancement scenario (right) upon harvest on day 15. Separation distances from left to right in each grouping were 45, 35, 25, and 15 cm. A gradient of pigmentation was evident in the yield-enhancement scenario.

On day 15, as the crop foliar canopy closed (**Figure 3**), end-point growth parameters including total shoot fresh and dry biomass were collected on an experimental unit basis of trays. kWh of energy expenditure for each LED lighting system over 15 days were recorded. Energy Utilization Efficiency (EUE) was calculated as grams fresh or dry biomass produced per kWh of electrical energy expended on the LED lighting system for all tested separation distances.

### 3.1 Energy-efficiency strategy

**Figure 4:**
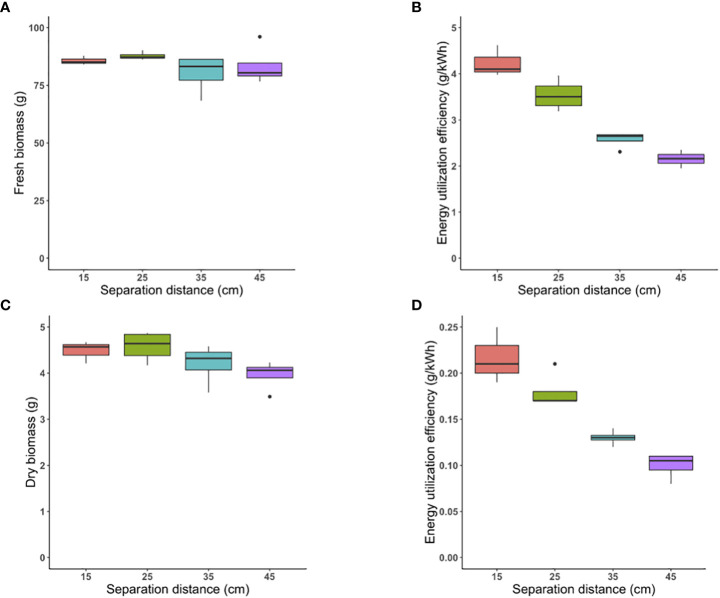
Energy-efficiency strategy: The fresh biomass produced per tray under control (45 cm) and three tested separation distances of 35 cm, 25 cm, and 15 cm **(A)**. The Energy Utilization Efficiency (g/kWh) of fresh biomass at all separation distances **(B)**. The dry biomass of plants grown per tray under control separation distance of 45 cm and three tested separation distance of 35 cm, 25 cm, and 15 cm **(C)**. The Energy Utilization Efficiency (g/kWh) of dry biomass at all separation distances **(D)**.

The same PPFD was provided (by selective dimming) under each separation distance tested; thus, the same biomass yield was expected with significantly different energy utilized at each separation distance. Using one-way ANOVA, statistical analysis confirmed that there was not a significant difference between fresh biomass of each group mean, considering an α level of 0.05 (p-value = 0.312) (**Figure 4A**).

kWh of energy expended on LED lighting systems was 40 over the entire 15-day cropping cycke for the control separation distance of 45 cm, and was 31, 25, and 20 kWh for the 35, 25, and 15-cm separation distances, respectively.

The EUE (ratio of gram fresh biomass produced per kWh of electricity consumed) was 2.1, 1.7, and 1.2-fold higher at the closest separation distance of 15 cm compared with 45 cm, 35 cm, and 25 cm, respectively (p-value<0.0001, p-value<0.000 and p-value=0.015, respectively) (**Figure 4B**).

Similarly, there was no significant difference between dry biomass of plants grown under the three tested separation distances compared to control considering an α level of 0.05 (p-value= 0.069) (**Figure 4C**).

Following a similar trend, EUE (ratio of gram dry biomass produced per kWh of electricity consumed) was 2.2-, 1.6-, and 1.2-fold higher at the closest separation distance of 15 cm compared with 45 cm, 35 cm, and 25 cm, respectively (p-value<0.0001, p-value<0.0001 and p-value=0.03, respectively) (**Figure 4D**).

### 3.2 Yield-enhancement strategy

**Figure 5:**
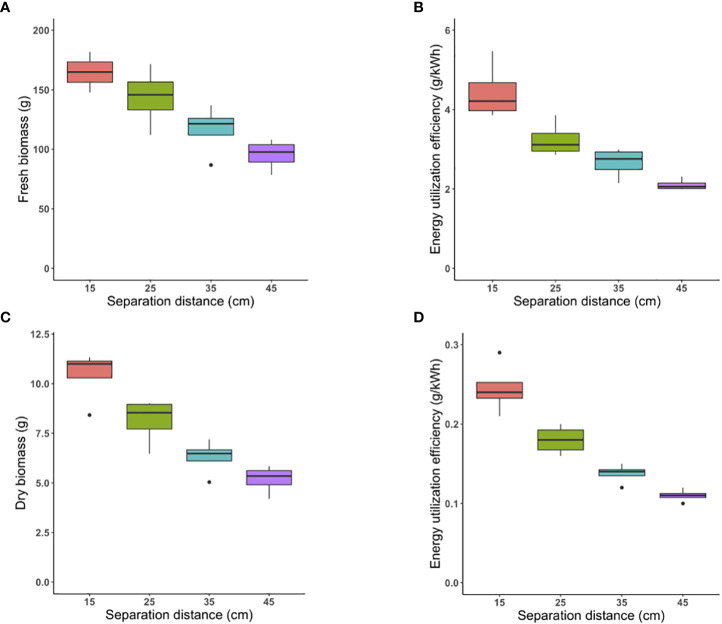
Yield-enhancement strategy: The fresh shoot biomass produced per tray under control (45 cm) and three closer separation distances of 35 cm, 25 cm, and 15 cm **(A)**. The Energy Utilization Efficiency (g/kWh) of fresh biomass at all separation distances **(B)**. The dry biomass of plants grown per tray under control separation distance of 45 cm and three tested separation distance of 35 cm, 25 cm and 15 cm **(C)**. The Energy Utilization Efficiency (g/kWh) of dry biomass at all separation distances **(D)**.

The LED lighting units were not dimmed at closer separation distances for this strategy; thus, significantly higher shoot biomass was expected while the same electrical energy would be utilized at each position. Based on one-way ANOVA, significantly higher fresh biomass was indeed produced under closer separation distances (p-value=0.0005) (**Figure 5A**).

Equivalent electrical energy was expended lighting all LED fixtures at different separation distances, with the average being 40 kWh. The EUE, defined as the ratio of g fresh biomass produced per kWh of energy consumed, was 2.1-, 1.7-, and 1.3-fold higher at the 15-cm separation distance compared with 45-cm, 35-cm and 25-cm separation distances, respectively (p-value<0.0001, p-value<0.0001 and p-value= 0.01, respectively) (**Figure 5B**).

Likewise, there was a highly significant difference in dry biomass at all tested separation distances (p-value<0.0001) (**Figure 5C**). As expected, the closest separation distance of 15 cm yielded the highest dry biomass as plants were exposed to the highest PPFD. The separation distance of 25 cm benefited from the second highest PPFD and resulted in higher yield compared to the other two greater distances. The standard separation distance of 45 cm had the least biomass, while biomass produced at the 35-cm separation distance was between the 25-cm and 45-cm treatments.

The EUE (ratio of gram dry biomass produced per kWh of electricity consumed) for the closest separation distance of 15 cm was 2.1-, 1.8-, and 1.3-fold higher than 45-cm, 35-cm, and 25-cm separation distances (p-value<0.0001, p-value<0.0001, and p-value=0.003, respectively) (**Figure 5D**). Consistent with the first scenario, by reducing the vertical distance between LED-emitting surface and foliar crop surface, EUE was highest for the least vertical separation distance of 15 cm, followed by 25 cm, 35 cm, and 45 cm.

### 3.3 Combination of close-canopy lighting and reflective curtain

#### 3.3.1 Energy-efficiency scenario & reflective curtain

**Figure 6:**
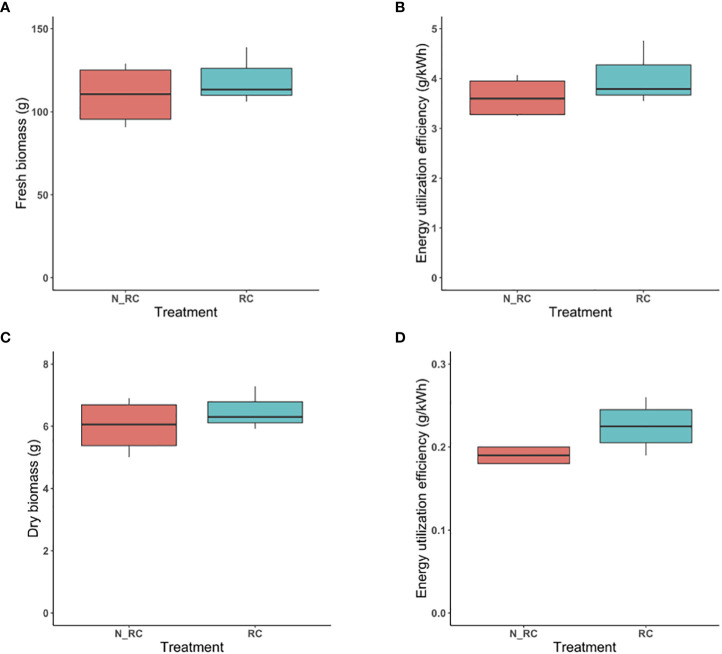
The combination of energy-efficiency strategy with or without reflective curtain for fresh biomass produced per tray at 25-cm separation distance **(A)**. The Energy Utilization Efficiency (g/kWh) of fresh biomass under those treatments **(B)**. Dry biomass produced per tray with or without curtains at 25-cm separation distance **(C)**. Energy Utilization Efficiency (g/kWh) of dry biomass under those treatments **(D)**. RC stand for “reflective” curtain” and N_RC stand for “no- reflective curtain.

Initial results indicated that although slightly higher fresh biomass was produced by the treatment with reflective curtain, there was no significant difference between fresh biomass produced under the combination of first CCL treatment and reflective curtain. Surrounding the growing system with a reflective curtain also did not result in higher fresh biomass (p-value=0.25) (**Figure 6A**). Since the same electrical energy for lighting was applied to both treatments, there was no statistically significant difference between EUE with or without curtains (p-value= 0.169) (**Figure 6B**). Dry biomass produced under those treatments also was not statistically different (p-value=0.22) (**Figure 6C**), and a similar trend was observed for EUE (p-value=0.079) (**Figure 6D**).

#### 3.3.2 Yield-enhancement scenario & reflective curtain

**Figure 7:**
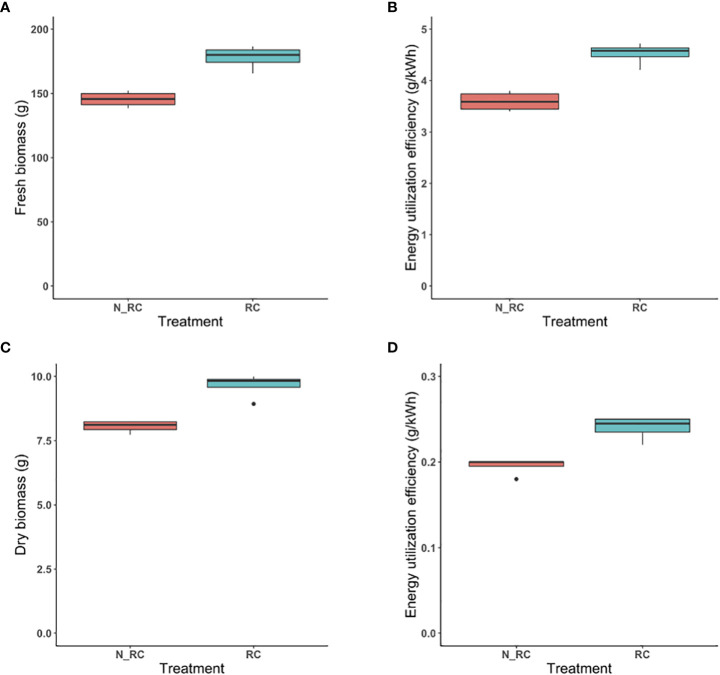
The effect of reflective curtain on CCL yield-enhancement strategy. Fresh biomass produced per tray with or without curtains at 25-cm separation distance **(A)**. Energy Utilization Efficiency (g/kWh) of fresh biomass under those treatments **(B)**. Dry biomass produced per tray under those treatments at 25-cm separation distance **(C)**. Energy Utilization Efficiency (g/kWh) of dry biomass under those treatments **(D)**. RC stand s for “reflective curtain” and N_RC stands for “no- reflective curtain”.

When a reflective curtain was used in combination with higher PPFD, significant increases in fresh biomass occurred compared to control (p-value=0.001) (**Figure 7A**). While the same energy was expended for LED lighting, higher biomass resulted in significantly higher EUE when the growth compartment was surrounded by reflective curtains (p-value=0.0007) (**Figure 7B**).

Likewise, dry biomass was significantly higher for the treatment with reflective curtain (p-value = 0.001) (**Figure 7C**) and significantly higher for EUE (p-value=0.002) (**Figure 7D**).

## 4 Discussion

LEDs currently available for indoor farming have much-improved electrical efficiency and photon efficacy compared to other plant-growth-lighting sources. The combination of significantly higher efficacy, extended lifetime, and relative lack of thermal radiation from photon-emitting surfaces make LED lighting systems the only real choice for sole-source indoor lighting.

However, LED physical capabilities can be further leveraged to enhance crop canopy photon capture efficiency (CCPCE) or utilance, efficiency metrics of the fraction of emitted photons incident upon photosynthetic surfaces of plants (Balasus et al., 2021). If CCPCE also is high, overall EUE of an indoor crop-lighting system can be further enhanced. Otherwise, LED efficiency and efficacy improvements are minimized by significant wastage of photons and energy.

In the present small-scale proof-of-concept study, we demonstrate enhanced CCPEC and EUE by implementing CCL strategies. Significant energy is saved as LEDs are dimmed at closer separation distances (energy-efficiency strategy), or significantly higher biomass is produced while the same energy is expended (yield-enhancement strategy). In support of our hypothesis, biomass produced at different separation distances was not significantly different in the energy-efficiency CCL strategy, although the closest separation distance tended to have higher biomass followed by the other three separation distances in order. LED beam-spread distribution was Lambertian with the highest light intensity at the center of a 120° beam. Although beam spread was fixed at various separation distances between emitting and absorbing surfaces, closer separation distances benefitted from the brighter center, which tended to have slightly higher fresh and dry biomass.

The present study highlights the need for deeper knowledge of the beam-spread pattern of LED lighting sources prior to making lighting maps for IF/VF. Better understanding in this particular area would enhance energy-use efficiency by saving significant energy expended on LED lighting systems. Upscaling of this study’s findings would reveal design challenges that need to be addressed.

Since LEDs as presently constructed typically follow Lambertian photon distribution, beam spread is wide, causing measured light intensity to decline with larger incremental distance between lighting source and illuminated surface due to photons that escape sensors (Wang et al., 2019). Thus, separation distance between photon-emitting surface area and photon-absorbing surface area matters.

At a standard vertical-farm separation distance of 40-50 cm between photon-emitting surface and light-absorbing crop surface, chance of escape is higher for photons emitted obliquely outward from LEDs positioned above or near the edges of cropping benches, but some escape is likely, even from LEDs positioned near the center of benches (**Figure 2**).

“Current droop” is a mechanism by which a significant loss of efficiency occurs from GaN-based LEDs (Piprek, 2010; Oh et al., 2019). As the forward current of LEDs increases, light output increases, but with a significant decrease of output efficiency. The current-droop phenomenon occurs as a result of an internal quantum-well-loss process, regardless of heat generation at the emitting surface (Laubsch et al., 2009). At ambient temperature, external quantum efficiency drops significantly at higher currents as a result of current droop (Meyaard et al., 2012). LED packages must operate at higher current to achieve desired intensity at farther separation distances, which in turn lowers efficiency. Therefore, reducing separation distance will improve photon absorption, as lower forward current can be applied to achieve the same PPFD. LED efficacy at reduced current density also can be improved by increasing the size of an LED chip (Kusuma et al., 2020). However, increasing chip size to reduce current droop specifically works for high-power LED devices (Meyaard et al., 2012), whereas most commercially available LEDs used for IF/VF are mid-power.

LED fixture design will be one important factor determining the success of close-canopy-lighting applications. Number of engines and their mounting patterns on a fixture will affect uniformity of both photon intensity and spectrum. Uniformity of intensity is impacted when cropping areas are affected by shade bands from other electrical components. A shade-band effect might not be pronounced under standard separation distances of 45-55 cm; however, it becomes more problematic at closer separation distances. A crop growing under shade-band areas would react by shade-avoidance syndrome, growing away from shade (Elkins & van Iersel, 2020), and marketability and quality are compromised. A reasonable blend of wavelengths is crucial to avoid specific photomorphogenic effects of individual wavebands on plant morphology.

At the beginning of the current study, aluminum tables with support frames for adjustable-height overhead LED lighting fixtures were constructed within a walk-in controlled-environment chamber. A commercial partner provided multi-waveband, dimmable LED fixtures that we mounted at various heights above the tables containing plant trays. Following preliminary CCL testing, difficulty was noted achieving expected results, including uniform plant growth and leaf pigmentation within trays at closer separation distances. Spectroradiometer readings indicated spacing of individual LEDs to be too wide apart to provide adequate beam overlap among individual LEDs at closer separation distances. However, already present in our laboratory from a previous project were LED arrays constructed to provide ground-based control experiments for the Veggie plant-growth unit being used to grow leafy greens for astronauts on the International Space Station, which already were mounted on height-adjustable scissors jacks. Light engines were arrayed densely and uniformly on the underside of those BPSE fixtures, the LEDs were dimmable by waveband, and spectroradiometer scans indicated uniform spectra at all lamp/crop separation distances between 45 and 15 cm, meaning that light-engine density and LED distribution were sufficient to provide adequate proof-of-concept testing for the scenarios of our CCL study. Not all LED arrays currently in use at standard 40- to 50-cm separation distances between cropping surfaces and LED-lighting fixtures in the indoor-farming industry may work for the energy-saving or yield-enhancing advantages of CCL. Lighting-system designers will have to weigh those advantages of CCL against costs of re-designing lamps, supports, and benches for productivity, profitability, and sustainability.

Secondary-optics installation is typically considered to improve light-intensity uniformity as the light distribution from an LED package without secondary-optic lenses falls under Lambertian type and lacks uniformity of intensity (Jiang et al., 2010; Nian et al., 2019). It also truncates beam spread and photon losses beyond the edges of growth areas would be less (Zhenrong et al., 2009). However, a higher number of engines need to be installed to reach the desired uniformity of intensity, and reflectance losses from secondary optics further add to lower efficiency and efficacy. Therefore, secondary-lens installation is not considered an efficient strategy compared with CCL.

Among different overhead LED fixtures, planar tube types are most commonly used in vertical farm settings (71%), followed by panel LEDs (21%), rounded LEDs (6%), and other types (2%) (Paucek et al., 2020). Regardless of fixture type, the LED luminaire/lighting width is either the same as or beyond bench width (**Figure 2**). The longer width of LED lighting structures that are common in current VF settings ensures light uniformity to bench edges and avoids gradients of crop growth, which otherwise would be drawbacks for automated harvest and marketability. Photon loss from cropping edges is inevitable as installing fewer tubes across benches is not the solution to the light-uniformity issue. In that setting, if saving energy is desired, CCL is the more effective strategy as the chance of photons escaping from growth compartments can be reduced.

In general, wider benches are not a grower’s desired solution as traditional lateral air flow and CO_2_ enrichment occur less efficiently at the center of benches.

As a supplement to CCL, deploying reflective curtains around growth compartments further improved efficiency of light utilization. Even at the closest CCL separation distances, some photons still escape the growth area and are wasted. Our findings suggest that the outcome of this approach is highly dependent upon light intensity, since reflective curtains were more effective for yield-enhancement scenario. Effectiveness of curtains was evaluated at the second-most efficient CCL separation distance of 25 cm to promote adequate air circulation. However, at higher separation distances, the chance of photon incidence on crop surfaces is higher with curtains, which might result in a different outcome.

Although a combination of CCL and reflective curtains should lead to the most robust energy-efficient production system, CCL will cause overhead space limitation for horizontal air movement across benches. Traditional air movement and thermal control, both of which are essential in a VF setting, will be disrupted, especially if curtains also enclose growth compartments. A considerable water-vapor boundary layer can accumulate above crop stands in stagnant air as a result of crop transpiration. Such humidity must be removed to enhance nutrient absorption and allow evaporative cooling. CO_2_ refreshment allowing continued photosynthesis is another factor requiring adequate air turnover above crop surfaces.

Although we did not encounter air-movement issues in a small-scale experimental growth-chamber setting, innovative engineering solutions will be needed to negate possible negative effects of combined CCL and reflective curtain installment in future vertical-farm lighting configurations. For this CCL proof-of-concept study, we did not develop a specific LED lighting system, but leveraged the use of height-adjustable LED lighting systems that were available in our laboratory. The height-adjustable property and reasonable uniformity of intensity and spectra enabled us to demonstrate proof of concept for two CCL strategies. We did not investigate the optimal spectral composition for indoor lettuce production; instead, a general lighting recipe mimicking white light was used.

The lawn-of-baby-greens production system developed for this study resulted in higher EUE as the planting distance between small seedlings was minimized and foliar-canopy closure occurred within a 15-day cropping cycle. Tip burn or other physiological disorders that might result from restricted air movement are non-issues for baby-stage lettuce. When CCL is tested for more mature stages of leafy greens, appropriate air movement will be become more essential and the need for engineering solutions necessary.

The EUE and yield-enhancement findings of this CCL proof-of-concept study can be of near-term benefit to indoor production of leafy greens. In addition to the need for re-design of light fixtures for appropriate light-engine mounting patterns and densities on the underside of LED light fixtures, manufacturers of vertical mounting racks need to re-design structural supports to address airflow constraints imposed by CCL. Current standard separation distances between the underside of light fixtures and the top of crops (40 – 50 cm) is adequate to support unconstrained horizontal movement of fresh air across benches needed for thermal and humidity control, as well as CO_2_ refreshment needed for rapidly photosynthesizing crop biomass. However, close separation distances (15 – 25 cm) may constrain horizontal air movement across benches, requiring innovative re-design involving controlled-velocity vertical air movement around lamps and through tray-support infrastructure (Zhang and Kacira, 2022). In fact, vertical air movement has been found to raise vapor-pressure deficit (reduce humidity) and prevent tip-burn disorder in lettuce (Ertle, 2023), allowing multiple advantages of implementing CCL. The authors encourage consideration of adjustable-height light supports above stationary crop supports at each tier within vertical cropping areas for flexibility of access and to accommodate different CCL needs of different crops or at different stages of crop production. Such advancements will help improve crop productivity and EUE, reduce OPEX for lighting, and improve profitability potential of the indoor-agriculture vertical-farming industry.

## Data availability statement

The raw data supporting the conclusions of this article will be made available by the authors, without undue reservation.

## Author contributions

FS: first author, major contribution. MB and RM: middle authors provided supporting services, LED hardware and interpretation of results. CM: last authorship, lab director. All authors contributed to the article and approved thesubmitted version.
